# MS Cortical Lesions on DIR: Not Quite What They Seem?

**DOI:** 10.1371/journal.pone.0078879

**Published:** 2013-11-11

**Authors:** Varun Sethi, Nils Muhlert, Maria Ron, Xavier Golay, Claudia A. Wheeler-Kingshott, David H. Miller, Declan T. Chard, Tarek A. Yousry

**Affiliations:** 1 Queen Square MS Centre, UCL Institute of Neurology, London, United Kingdom; 2 Department of Neuroinflammation, UCL Institute of Neurology, London, United Kingdom; 3 Department of Brain Repair and Rehabilitation, UCL Institute of Neurology, London, United Kingdom; 4 Lysholm Department of Neuroradiology, National Hospital for Neurology and Neurosurgery, London, United Kingdom; Mayo Clinic, United States of America

## Abstract

**Objective:**

Accurate identification and localization of cortical gray matter (CGM) lesions in MS is important when determining their clinical relevance. Double inversion recovery (DIR) scans have been widely used to detect MS CGM lesions. Phase sensitive inversion recovery (PSIR) scans have a higher signal to noise, and can therefore be obtained at a higher resolution within clinically acceptable times. This enables detection of more CGM lesions depicting a clearer cortical and juxtacortical anatomy. In this study, we systematically investigated if the use of high resolution PSIR scans changes the classification of CGM lesions, when compared with standard resolution DIR scans.

**Methods:**

60 patients [30 RR(Relapsing remitting) and 15 each with PP(Primary progressive) and SP(Secondary progressive) MS] were scanned on a 3T Philips Achieva MRI scanner. Images acquired included DIR (1×1×3 mm resolution) and PSIR (0.5×0.5×2 mm). CGM lesions were detected and classified on DIR as intracortical (IC) or leucocortical (LC). We then examined these lesions on corresponding slices of the high resolution PSIR scans and categorized them as IC, LC, Juxtacortical white matter (JC-WM, abutting but not entering cortex) and other white matter (WM, not juxtacortical). Classifications using both scans were noted.

**Results:**

282 IC and 483 LC were identified on DIR. Of the IC lesions, 61% were confirmed as IC on PSIR, 35.5% were reclassified as LC and 3.5% as JC-WM or other WM only. Of the LC DIR lesions, 43.9% were confirmed at LC on PSIR, 16.1% were reclassified as IC and 40% as JC-WM or other WM only. Overall, 50% (381/765) of CGM lesions seen on DIR were reclassified, and 26.5% (203/765) affected WM only.

**Conclusions:**

When compared with higher resolution PSIR, a significant proportion of lesions classified as involving CGM on DIR appear to either contain more white matter than expected or to not involve CGM at all.

## Introduction

Recent advances in imaging techniques have allowed the *in vivo* detection of multiple sclerosis (MS) cortical gray matter (CGM) lesions which were previously only observed histopathologically. Double inversion recovery (DIR) [1]–[4] has been most extensively used to study CGM lesions *in vivo*, and it has been shown that accrual of CGM lesions relates to disability in established MS [2], [4], [5] and their presence may also improve the specificity of current MS MRI diagnostic criteria [1]–[3]. However, a recent combined histopathology and MRI study has shown that even with the use of 3D DIR only 17% CGM lesions were detected prospectively [6].

MS cortical GM lesions have been histo-pathologically sub-classified based on (i) location i.e. relative to GM/CSF and GM/WM boundary and (ii) morphology. Based on their location they have been sub-classified as abutting the cerebrospinal fluid (CSF) but not extending through the whole cortical ribbon, traversing the full thickness of the cortex from the CSF to WM interfaces, or confined to the centre of the cortex (not touching either the CSF or WM boundaries) [1], [2], [4], [7], [8].

Using the standard resolution 2D DIR sequences that have been acquired in many published studies of MS to date [1], [3], [4], [8]–[12], it is difficult to reliably identify subtypes of GM lesions [1]–[4]. In particular, juxtacortical WM lesions (JC-WM) - one of the subtypes of WM lesions that count towards MS MRI diagnostic criteria [2], [4], [5] have the potential to be confused with mixed GM-WM lesions. Due to the intrinsically low signal to noise of DIR, it is difficult to increase the resolution beyond that already obtained while maintaining clinically acceptable scan times. In contrast, phase sensitive inversion recovery (PSIR) scans can be acquired at significantly higher resolution within clinically feasible times, and recent work has suggested that on higher resolution PSIR scans it is possible to detect about three times as many CGM lesions. In addition, when compared with standard resolution DIR, the cortical ribbon and adjacent WM are more distinct on PSIR, allowing juxtacortical WM (JC-WM) lesions to be separated from mixed GM-WM lesions [3]. This raises the possibility that some lesions identified as purely or partly involving CGM on DIR may actually contain WM, or be entirely within the juxtacortical WM. This has important implications when considering the clinical relevance of CGM relative to JC-WM lesions, and it is possible that a significant proportion of lesions classified as CGM on DIR may contain substantially more WM than expected.

CGM lesions have also been divided into subtypes based on morphology, on DIR [1]–[3] and PSIR [1], [2], [4], [6], [7]. More recent work has suggested that curvilinear lesions may help differentiate age-associated GM lesions from those due to neuroinflammation [1], [2], [4], [7], [8].

We hypothesized that the higher resolution of PSIR will improve the classification of CGM lesions beyond that possible using standard resolution 2D DIR in clinically acceptable scanning times. A better understanding of the extent and location of gray matter involvement and possibly the morphology of lesions will improve the specificity of observations made in the context of MS and reduce the noise while interpreting correlations between CGM lesion and parameters of cognitive and clinical parameters.

Different groups have investigated the roles of DIR [4], [8], [9], PSIR [1], [3], [10]–[12] and a combination of DIR and PSIR in improving lesion detection; [13], [14]. However, an *in vivo* assessment of the accuracy of CGM lesion localization using the widely reported standard 2D DIR sequence has not been previously undertaken. Having acquired high resolution PSIR and standard resolution DIR images in a cohort of 60 MS patients, we now compare the location of CGM lesions defined on DIR with their location defined on the higher resolution PSIR images that provide a considerably improved anatomical definition of the cortex and adjacent white matter.

In this study, we determined if lesions seen on DIR images were similarly classified in terms of location and morphology on higher resolution PSIR scans.

## Materials and Methods

### 1.1 Participants

A cohort of 60 patients {30 relapsing-remitting (RR) MS, 15 each with primary (PP) and secondary progressive (SP) MS} had been recruited for earlier work that showed a quantitative improvement in CGM lesion detection using PSIR (vs. 2D DIR). [1]. We now studied this cohort to investigate the change in classification of lesions across both these scans This work was reviewed and approved by the NRES Committee London-Queen Square. All patients gave written informed consent.

### 1.2 Image Acquisition

DIR, PSIR and fluid attenuated inversion recovery (FLAIR) scans were acquired on a 3T Achieva TX system (Philips healthcare, Best, The Netherlands) with a 32-channel coil(Table 1).

**Table 1:** Acquisition parameters.

	Resolution (mm)	FOV (mm^2^)	TR (ms)	TE (ms)	TI (ms)	Slices	SENSE	Time (mins)
***FLAIR***	1×1×3	240×180	8000	125	2400	50	1.3	3.4
***DIR***	1×1×3	240×180	16000	9.9	3400/325	50	2	4.16
***PSIR***	0.5×0.5×2	240×180	7306	13	400	75	–	11.26

FOV = field of view, TR = repetition time, TE = echo time, TI = inversion time, SENSE = sensitivity encoding factor.

### 1.3 Image Analysis

CGM lesions were identified and classified independently on DIR, using FLAIR as a reference when needed. Lesion marking and sub-classification was performed as per published guidelines [1], [8]).

CGM lesions were first identified and localized on DIR. We then identified the same lesions, on corresponding slices of the high resolution PSIR scans and classified them based on their location as it appeared on the PSIR scan. Classifications on both DIR and PSIR were noted. All lesions were marked under supervision of senior raters (including an experienced neuro-radiologist [TY]). Intra-rater and Inter-rater reproducibility for this process has been shown to be good in earlier published work. [1].

On DIR, the lesions were classified as intracortical (IC, only involving GM) or leucocortical (LC; mixed GM-WM lesions). JC-WM lesions could not be separated from the adjacent cortex using DIR, but these could be distinguished using PSIR, owing to the improved grey –white contrast and better SNR, on the higher resolution PSIR. The CGM lesions detected on DIR (IC or LC) were thus classified on PSIR scans in to one of 4 categories: IC, LC, JC-WM lesions (WM lesions abutting but not entering the cortex), or other WM lesions (non-JC). Based on their morphology, cortical lesions were sub-classified by shape as curvilinear (lesions that follow the contour of sulcal and gyral folds), oval or wedge shaped [2].

Classification of DIR visible CGM lesion location lesions was first performed and the noted using DIR sequence and then – for each lesion, the corresponding classification on PSIR was noted. We noted changes in classification from either IC/LC on DIR to IC/LC/JC or WM on PSIR.

## Results

**Figure 1:**
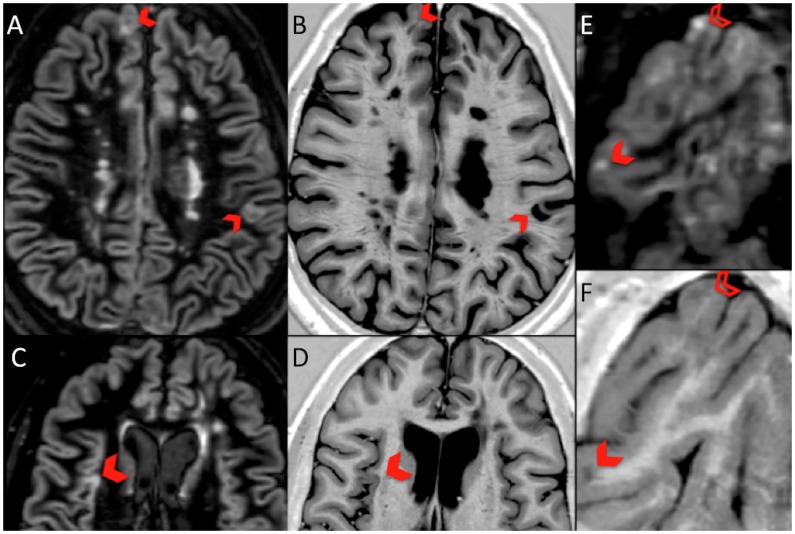
Corresponding DIR and PSIR images showing change of classification of CGM lesions. DIR LC lesions (blocked chevron) in panel A, appear as JC-WM lesions on PSIR in panel B; DIR LC lesion in panel C is seen to be a pure IC lesion on PSIR in panel D; DIR LC lesion (blocked chevron) and IC lesion (open chevron) in panel E appear as JC WM and LC on PSIR respectively, in panel F.

765 CGM lesions were marked on DIR, 282 of these were IC and 483 LC. Of the 282 IC lesions, 172 (61%) were confirmed as IC on PSIR, and 110(39%) were reclassified (100 LC [35.5%], 8 JC-WM [2.8%] 2 Non-JC WM (0.7%)). Of the 483 LC DIR lesions, 212 (43.9%) were confirmed as LC on PSIR, and 271 (56.1%) were reclassified (78 IC [16.1%], 155 JC-WM, 38 WM [together totaling 40%]). (Table 2; Figure 1).

**Table 2:** Change of classification of lesions from DIR to PSIR (based on lesion type).

		*As subsequently seen on PSIR*			
		*IC*	*LC*	*JC*	*WM*
***On DIR***	***IC (N = 282)***	172(61)	100(35.5)	8(2.8)	2(0.7)
	***LC (N = 483)***	78 (16.1)	212 (43.9)	155(32.1)	38 (7.9)

[number (percentage)] Each row corresponds to a lesion type seen on DIR, and each column how the same lesion was classified on PSIR. E.g. of the IC lesions so classified on DIR, 60% remained so on PSIR.

Morphologically, on DIR, 665 of CGM lesions were initially classified as oval, 59 curvilinear and 41 wedge shaped. Using PSIR 148/765 (19.3%) were reclassified (see Table 3). The most striking difference was the higher number of lesions classified as curvilinear on PSIR than on DIR.

**Table 3:** Change of classification of lesions from DIR to PSIR (based on morphology of lesions).

		*As subsequently seen on PSIR*		
		*Oval*	*Wedge*	*Curvilinear*
***On DIR***	***Oval (N = 665)***	523 (78.6)	26 (3.9)	116 (17.4)
	***Wedge (N = 41)***	0	30 (73.2)	11(26.8)
	***Curvilinear (N = 59)***	5 (8.5)	0	54 (91.5)

[number (percentage)] Each row corresponds to a lesion type seen on DIR, and each column how the same lesion was classified on PSIR. E.g. of the IC lesions so classified on DIR, 60% remained so on PSIR.

## Discussion

In this study we found that MS lesions reported to involve the CGM on DIR were often reclassified in a different anatomical location when viewed on a higher resolution PSIR sequence. Specifically, 39% (110/282) lesions thought to only involve CGM on DIR (i.e. IC lesions) where noted to extend into WM on PSIR, and of the mixed GM-WM lesions on DIR (i.e. LC) about 56% (271/483) were reclassified on PSIR, with about 40% (193/483) classified as being purely in the WM.

A recent combined histopathological and MRI study reported that 90% of CGM lesions seen on a high resolution 3D DIR scan (using a scan resolution of 1.1 by 1.1 by 1.3 mm, which is higher than those used in the present study and that have commonly been used in previous *in vivo* studies) were histopathologically confirmed [6]. However, the present study suggests that at the resolution that has been more often employed in clinical DIR studies published to date (1×1×3 mm) a larger proportion of DIR identified CGM lesions may actually be false positives.

In a previous study where CGM lesions were marked independently on DIR and high resolution PSIR, about three times more CGM lesions were detected on PSIR [1]. However, the high frequency in the present study with which DIR CGM lesions were reclassified as WM lesions suggests that the increase in true CGM lesion detection on PSIR may be even higher, perhaps about 6 times that of DIR.

Not surprisingly, DIR-classified LC lesions were more likely than IC lesions to be reclassified as being entirely in the WM (40% and 3.5% respectively). JC-WM lesions seen on conventional T2-weighted and FLAIR sequences are included in the current McDonald 2010 diagnostic criteria [5] but it has been suggested that intracortical lesions seen on DIR may also have a role in improving the accuracy of MRI diagnostic criteria for MS. [3], [9]. Noting that a significant proportion of lesions classified as involving CGM on DIR may actually be JC WM lesions, this raises the question of whether more accurate classification of CGM lesions would enhance their role in diagnostic criteria. Prospective studies following CIS patients who have had high resolution PSIR and DIR scans at presentation could address this question. From this work we also cannot determine if it is resolution or contrast that most influences the classification of lesions on DIR or PSIR. Pragmatically, it is not currently possible (due to intrinsically low signal to noise of DIR) to increase the resolution of DIR to match that of the PSIR scan used in this study while remaining within clinically acceptable scan times.

Cognitive impairment is seen in about 40–60% patients with MS [15], [16] and previous work using standard resolution DIR has demonstrated correlations between cortical lesion load and measures of cognitive and clinical disability [14]. These data support a role for lesions in or near the cortex in determining clinical outcomes [14]. A higher cortical lesion number has been reported in those MS patients who are cognitively impaired and cortical lesion volume has been found to be an independent predictor [17]. Further, a study using FLAIR scans reported an association of cognitive impairment with JC-WM lesions [18]. Thus, accurate classification and quantification of CGM and JC-WM lesions is key in studying whether this impairment is driven by pathology in the cortex, juxtacortical WM or both regions. The potentially limited accuracy of CGM classification on DIR again raises questions about the actual contribution of CGM and JC-WM lesions to physical and cognitive deficits, and this maybe worth revisiting with a combination of methods such as high resolution PSIR and DIR.

Consensus recommendations for marking CGM lesions on DIR were published in 2010 [8]. While both 2D DIR and 3D DIR have improved the detection of cortical lesions, PSIR (voxel size 0.5 mm^3^; 0.5×0.5×2 mm) provides a greater resolution than both the commonly used 2D DIR (voxel 3 mm^3^; 1×1×3 mm i.e. Six times higher] and 3D DIR (voxel 1–2 mm^3^) [2–4 times higher]. DIR has a low SNR. Further, as scan times are proportional to the inverse of the square root of SNR, any attempt to get similar sub-millimeter in plane resolution and to maintain the same SNR using DIR will take a prolonged time that is usually not acceptable for clinical purposes. It is for this reason that a direct comparison with both scans at a similar resolution is not practical in a clinical setting. PSIR has a higher SNR and provides an improved anatomical definition with high in-plane resolution (0.5×0.5 mm) in clinically acceptable times. [1] Nevertheless, there are now widely available 3D DIR sequences that have better resolution than the standard 2D DIR sequence used in the present study and a comparison of these sequences with high resolution PSIR could usefully be undertaken.

In our earlier study, we identified curvilinear lesions in 36% of people with MS using DIR and in 85% using PSIR, while no such lesions were seen in healthy controls [3]. Investigation is required in people with other neurological conditions to determine the specificity of such lesions for MS, but these findings do suggest a potential role in diagnosis. Given differences in the apparent frequency of curvilinear lesion on DIR and PSIR, we looked at the consistency of CGM lesion morphological classification between DIR and PSIR, and found that 27% of the wedge shaped and 17% of the oval shaped lesions were reclassified as curvilinear on PSIR. This suggests that, at least in part, the apparently higher number of curvilinear lesions seen on PSIR when compared with DIR may be due to greater accuracy. Given the contrasting finding between patients and controls, the lesion morphology may also help in the diagnosis of MS when it is suspected but not confirmed.

In conclusion, we have found that a significant proportion of lesions thought to involve CGM on standard resolution DIR images are reclassified as WM lesions when viewed on a higher resolution PSIR sequence. While DIR has improved cortical lesion detection [4], [9], [10], in addition to increasing the number of CGM lesions identified, PSIR is also likely to improve the accuracy of CGM lesion classification.

In future a multimodal approach, using multiple scans like DIR (especially higher resolution 3D DIR sequences) and PSIR, may help with CGM lesion detection and localization [13]; PSIR however provides useful additional information regarding the classification of lesion location and morphology. The findings of this work warrant attention in future studies investigating the role of CGM lesions in MS diagnostic criteria and in investigating how CGM and adjacent WM lesion location and morphology relate to cognitive or neurological function. While post mortem verification of cortical lesions using DIR has recently been reported [6], [19], a similar post mortem study to PSIR visible cortical lesions should provide an improved understanding of our *in vivo* results.
